# The Choice of Pedometer Impacts on Daily Step Counts in Primary School Children under Free-Living Conditions

**DOI:** 10.3390/ijerph16224375

**Published:** 2019-11-09

**Authors:** Chiaki Tanaka, Yuki Hikihara, Shigeru Inoue, Shigeho Tanaka

**Affiliations:** 1 College of Health and Welfare, J. F. Oberlin University, Tokyo 194-0294, Japan; 2 Yuki Hikihara, Chiba Institute of Technology, Chiba 275-0023, Japan; hikihara.yuki@it-chiba.ac.jp; 3 Department of Preventive Medicine and Public Health, Tokyo Medical University, Tokyo 160-8402, Japan; inoue@tokyo-med.ac.jp; 4 Department of Nutrition and Metabolism, National Institute of Health and Nutrition, National Institutes of Biomedical Innovation, Health and Nutrition, Tokyo 162-8636, Japan; tanakas@nibiohn.go.jp

**Keywords:** physical activity, children, acceleration

## Abstract

*Background*: We examined whether daily step counts under free-living conditions differed among four types of pedometers used by primary school children. *Methods*: In Study one, we compared the Yamax SW-200 (widely used in research) and the Kenz Lifecorder (accelerometer-based pedometer) in 30 children (6–12 years). In Study two, after confirming good correlation between these devices, we used Kenz Lifecorder as the criterion device and compared it with the Yamasa EX-200 (pants pocket-type pedometer) and the Omron Active style Pro (accelerometer-based pedometer) among 48 (7–12 years) or 108 children (7–12 years). *Results*: In Study one, comparable mean step counts between pedometers were observed. The correlation was strong (*r* = 0.91); the average difference between these two pedometers was +4.5%. In Study two, the average differences between Kenz Lifecorder and Yamasa EX-200 and Kenz Lifecorder and Omron Active style Pro were −7.9% and −18.2%, respectively, and those were not significantly equivalent according to the two one-sided-tests method. The correlations between Yamasa or Omron Active style Pro and Lifecorder were moderate and strong, respectively. *Conclusions*: The choice of pedometer had a substantial impact on step counts. A consensus on the appropriate pedometer for quantifying daily step counts is needed for evidence-based recommendations for health promotion.

## 1. Introduction

Physical activity guidelines for young people have been available since the 1980’s. Currently, most of these guidelines recommend at least 60 minutes of moderate-to-vigorous-intensity physical activity per day [1]. Tudor-Locke et al. [2] reviewed the existing literature on objectively monitored step-defined physical activity in children to provide researchers, practitioners, and lay people who use accelerometers and pedometers with an evidence-based translation of these public health guidelines in terms of steps/day. They showed that 60 minutes of moderate-to-vigorous-intensity physical activity in primary/elementary school children appears to be achieved, on average, within a total range of 13,000 to 15,000 steps·day^−1^ in boys and 11,000 to 12,000 steps·day^−1^ in girls. “Steps per day” is a very familiar physical activity index, not only for researchers, but also for the general public, and ultimately is more likely to be embraced by the public, due primarily to ease of interpretation and relatively low cost. Nationwide surveys in the United States [3], Canada [4] and Japan [5] use pedometer-based monitors to evaluate physical activity, while health promotion campaigns often use pedometers to encourage physical activity in children and adults [6]. In fact, a strong correlation has been reported between minutes of moderate-to-vigorous physical activity and step counts (*r* = 0.832, *p* < 0.001) [7].

Several studies have reported on the accuracy of pedometers in children [8,9,10,11], in which the actual steps counted using a hand-tally counter were compared with values obtained by pedometers. The data showed a large variation in counts during slow-pace walking, but not during normal and fast-pace walking. For example, Nakae et al. [10] compared the accuracy of the Yamax EC-200 pedometer with 2 accelerometer-based (Kenz Lifecorder and Omron HJ-700IT) pedometers in laboratory settings in primary school children. However, it is important to know to what extent differences in daily step counts measured by various types of pedometers vary under free-living conditions in children. We identified only one study that compared the Lifecorder accelerometer-based pedometer with the Yamax SW-200 pedometer and the ActiGraph accelerometer with an activated step count function under free-living conditions in 10-year-old children in the United States [12]. The Lifecorder and the Yamax SW-200 detected a comparable number of steps, similar to the results in adults published by Schneider, Crouter, and Bassett [13].

It is important to know to what extent daily step counts by various types of pedometers differ under free-living conditions in children [12,14,15]. Although accelerometer-based pedometers, including piezo-electric pedometers, can be more accurate and precise, they are more expensive, and accordingly, spring-levered pedometers have often been used in large-scale investigations and interventions [14]. Recently, pedometers that can be placed in the participant’s pants pocket have become available and are more convenient than those attached on the hip. A physical activity investigation in a Tokyo metropolitan area for students including primary school children (n = 9858) was performed using a pants pocket-type pedometer (Yamasa, EX-200) [15]. Recently, the Active style Pro HJA-350IT accelerometer (Active style Pro; Omron Healthcare Corp., Tokyo, Japan), which was released for sale in Japan in 2008, and can measure step counts and evaluate sedentary behavior and light-to-vigorous physical activity intensity, was validated for Japanese primary school children (boys: 10.0 ± 1.7 years, girls: 9.0 ± 1.8 years) [16]. The Active style Pro has a user-friendly software interface that is easy to initialize and download, and the data can be downloaded and the steps within the reported time intervals summed (built-in memory). Therefore, many researchers in Japan have begun using the Active style Pro. In numerous studies for children, the Yamax digi-walker SW-200 has been used, and from the obtained values, standards of step counts for children have been established [2]. In addition, the Lifecorder has become a popular pedometer in research and can evaluate step counts better than others at a slow walking pace in laboratory settings. Considering such situations, evidence is necessary to determine whether utilization of those different pedometers for children influences the measurement of step counts under free-living conditions.

The purpose of this study was, therefore, to examine whether daily step counts in free-living conditions in children differed when measured with different pedometers. In Study one, we compared the criterion pedometer (the Yamax digi-walker SW-200: Yamax), a widely used pedometer in research studies, with an accelerometer-based pedometer (the Kenz Lifecorder EX: Kenz) with a user-friendly software interface and built-in memory. Moreover, in Study two, we compared Kenz Lifecorder with a pants pocket-type pedometer (the Yamasa EX-200: Yamasa) and the Active style Pro HJA-350IT (Omron Healthcare). The study also had the objective of determining equations to convert values from one pedometer to those of another pedometer.

## 2. Materials and Methods 

The participants were 138 Japanese primary school children (53 boys and 85 girls) ages 6 to 12 years (mean age, 9.3 ± 1.7 years) living in the Tokyo metropolitan area and Ibaragi prefecture. All of the participants reported being in good health, without any history of conditions that may affect energy expenditure, such as abnormal thyroid gland function. Informed consent was obtained from both a parent and the children themselves. The study protocol was approved by the Ethical Committee of J. F. Oberlin University (No. 10007). 

### 2.1. Study One: Kenz Lifecorder EX Compared to Yamax SW-200

Step counts in 30 participants obtained with Kenz Lifecorder Ex (Suzuken Corp., Nagoya, Japan) were compared with steps measured by Yamax SW-200 (Yamax Corp., Tokyo, Japan) which were worn on participants’ hip at the mid-line of the right and left thighs, respectively. The position (right/left) of the devices was not randomized. Yamax SW-200 was the criterion device, because it has been widely used in previous studies, including comparison or validation studies for children under free-living and laboratory settings [12,17]. However, Yamax SW-200 requires the daily step counts to be recorded before bedtime each day by either the participant or their parents. Kenz Lifecorder EX has been widely used also, and it has a user-friendly software interface and built-in memory. In previous review, a significant seasonal variation in physical activity for children was reported in all UK studies, being highest in summer and lowest in winter [18], although in non-UK studies a seasonal variation in physical activity was not found. Therefore, no measurements weren’t conducted in winter season. 

### 2.2. Study Two: Yamasa EX-200 and Active style Pro Compared to Kenz Lifecorder EX

In different samples from Study one, we compared Kenz Lifecorder and Yamasa EX-200 (Yamasa Corp., Nagoya, Japan) as well as Kenz Lifecorder and Omron Active style Pro HJA-350IT (Omron Healthcare Corp., Tokyo, Japan), which can measure step counts as an accelerometer-based pedometer, as stated above. For Kenz Lifecorder and Omron Active style Pro, with a computer interface, the data can be downloaded and the steps within the reported time intervals summed (built-in memory). Therefore, Kenz Lifecorder and Omron Active style Pro were then compared in 108 participants. On the other hand, our research staff had to record the daily step counts from Yamasa EX-200 for each participant immediately after the measurement period, because the device can remember and display the data for only 7 days, and the data cannot be downloaded. Therefore, only 48/108 participants wore Yamasa EX-200. The 48 children who wore the Yamasa pedometer were chosen randomly. The position (right/left) of the devices was not randomized. On the next day just after the measurement period, our research staff recorded the daily step counts from Yamasa EX-200 and uploaded data from Lifecorder and Omron Active style Pro. Therefore, data from 6 days were used for analyses. Activity record was used to determine non-wear days. To minimize an “order” effect, the participants attached either Kenz Lifecorder and Omron Active style Pro at the mid-line of the right and left thighs, and the subgroup (*n* = 48/108) put Yamasa EX-200 into their pants pocket for seven days, which included 5 weekdays and 2 weekend days. The participants were requested to wear these devices at all times, with the exception of special circumstances, such as dressing and bathing. No measurements weren’t conducted in winter season.

### 2.3. Anthropometry 

Height and body weight were measured to the nearest 0.1 cm and 0.1 kg, respectively. Height and body weight were measured in subjects without shoes by a stadiometer or a weighing scale at each school, according to the School Health Law by which all students in Japan are measured annually. Net body weight was calculated as the weight of clothing subtracted from the measured body weight. Clothing for each subject included uniform shorts and t-shirts, corresponding to a 0.5 kg deduction. In the 30 participants who wore Yamax SW-200 and Kenz Lifecorder, these parameters were self-reported. Body mass index (BMI) was calculated as body weight (kg) divided by squared body height (m). 

### 2.4. Analyses 

The data from 6 days, excluding days when the participants started wearing and removed pedometers, were used for analyses. In addition, there were missing data for some participants. As a result, full week data were not obtained (data were obtained from 4 or less weekdays and 1 or 2 weekend days). Participants lived on a weekly basis with 2 weekend days. Therefore, the average number of step counts for weekdays and weekend days were calculated for each individual, respectively, and then the average weekly value was calculated by weighting for 5 weekdays and 2 weekend days (Weighted data = (Average for weekdays × 5 + average for weekend days × 2)/7). As Rowe et al. [19] suggested that values below 1000 steps·day^−1^ and above 30,000 steps·day^−1^ should be treated as missing data, we excluded such data from the analyses. 

The data for this study are not publicly available but may be shared upon request. For further information on the data and materials used in this study, please contact the corresponding author.

The data were tested using the Shapiro-Wilk test for normal distribution prior to the main analysis. They showed a normal distribution. The percentage difference between Yamax SW-200 and Kenz Lifecorder was calculated as: ([steps for the Lifecorder − steps for the Yamax SW-200]/steps for the Yamax SW-200) × 100. The percentage difference between Kenz Lifecorder and each device was calculated as: ([steps for the device − steps for the Lifecorder]/steps for the Lifecorder) × 100. Moreover, mean absolute percent error (MAPE) for each device comparison was calculated as the mean of absolute differences between the two devices divided by the value of the criterion device. Pearson moment–product correlation coefficients (r) were examined to determine if there was a significant correlation between devices. Pearson moment–product correlation coefficients were also examined with adjustment for BMI. Comparisons of the step counts obtained with Kenz Lifecorder with those obtained with each device were assessed using Bland & Altman limits of agreements. A simple linear regression analysis was used to obtain the equations to convert the number of steps obtained with one pedometer to the number of steps that would be obtained with another. Two-one-sided-tests method was applied to provide evidence of equivalence. T value was calculated as: (steps for the Lifecorder − steps for the Yamax SW-200 − equivalence region)/(standard deviation of those difference) × 100 or (steps for the device − steps for the Lifecorder − equivalence region)/(standard deviation of those difference). The equivalence region was set as ±1000 steps/day, with the 90% confidence interval (α = 0.05) [20]. All results were expressed as mean ± SD (standard deviation). The statistical analyses were performed using SPSS version 23.0J for Windows (SPSS Inc, Japan, Tokyo). All statistical tests were regarded as significant when *p*-values were <0.05. 

## 3. Results

The mean height, weight, and BMI of the participants in Study one (*n* = 30) were 136.4 ± 13.7 cm, 33.6 ± 13.6 kg and 17.4 ± 3.7 kg·m^2^, respectively, and those for the participants on Study two (*n* = 48 and 108) were 133.8 ± 9.6 cm, 29.1 ± 6.6 kg, and 16.0 ± 1.9 kg·m^2^, and 133.4 ± 9.9 cm, 29.6 ± 6.9 kg, and 16.4 ± 2.0 kg·m^2^, respectively. Table 1 shows the daily step counts for each device and the percentage difference for each paired comparison. According to the two-one-sided-tests method, the 90% confidence interval of (−18, 938) was completely inside the equivalence region of (−1000, 1000), which indicates that steps measured by Kenz Lifecorder were significantly equivalent to steps measured by Yamax SW-200, with the average difference being 4.5 ± 13.4% and MAPE being 10.1 ± 9.7%. A strong correlation was observed between steps measured by Kenz Lifecorder and Yamax SW-200 (*r* = 0.910, *p* < 0.001, confidence interval: 0.818–0.957). All of these associations remained significant after adjusting for BMI. On the other hand, steps measured by Kenz Lifecorder were not significantly equivalent from steps measured by Yamasa EX-200 and Omron Active style Pro according to the two-one-sided-tests method (the 90% confidence intervals of (−1690, −806) and (−2786, −2389) were not inside the equivalence region for Yamasa EX-200 and Omron Active style Pro, respectively), with the average difference being 7.9 ± 21.3% and 18.2 ± 10.1% and MAPE being 14.8 ± 17.1% and 19.5 ± 7.3%, respectively.

**Figure 1:**
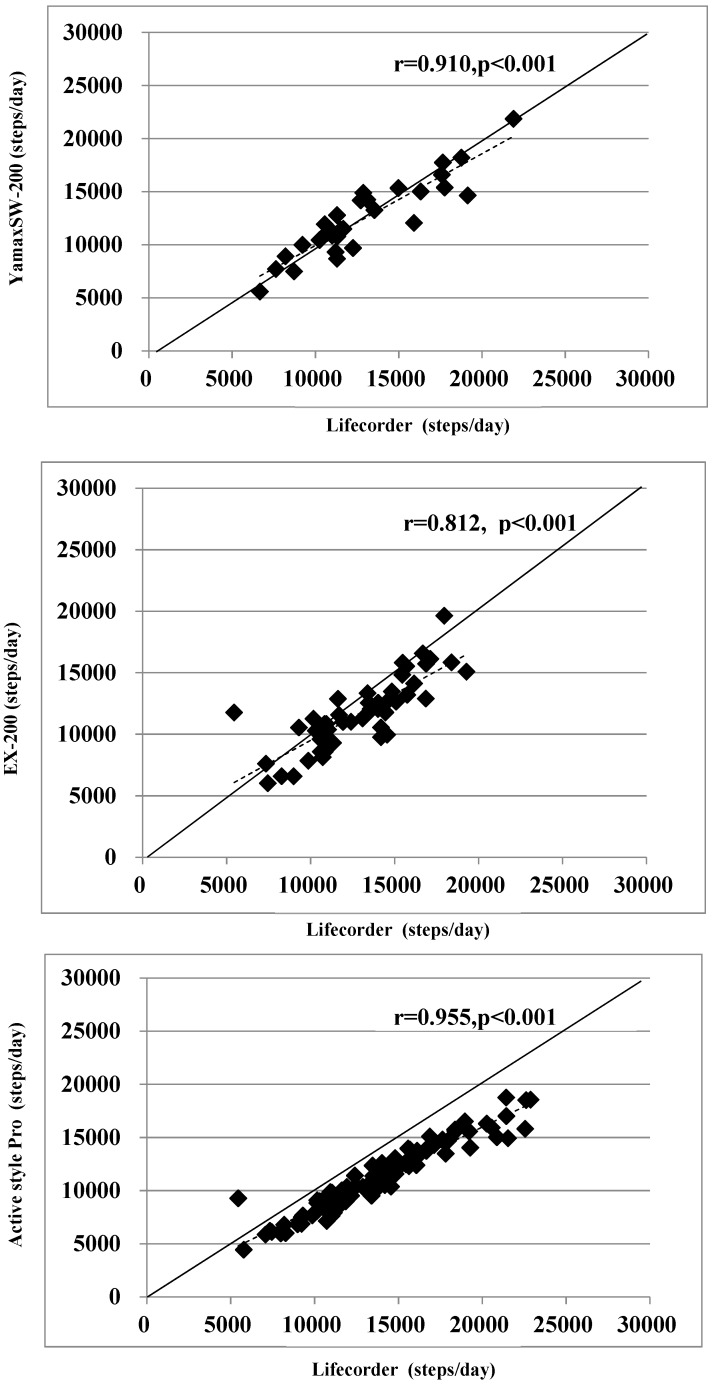
Comparison of step counts recorded by the four pedometers (solid line: identity line, dotted line: regression line).

Figure 1 shows the correlations for all of the devices. Pearson correlation coefficients showed significant associations between each device under free-living conditions. There was a moderate or strong correlation between Kenz Lifecorder and Yamasa EX-200 (*r* = 0.812, *p* < 0.001, confidence interval: 0.686–0.891) or Omron Active style Pro (*r* = 0.955, *p* < 0.001, confidence interval: 0.935–0.969).

**Figure 2:**
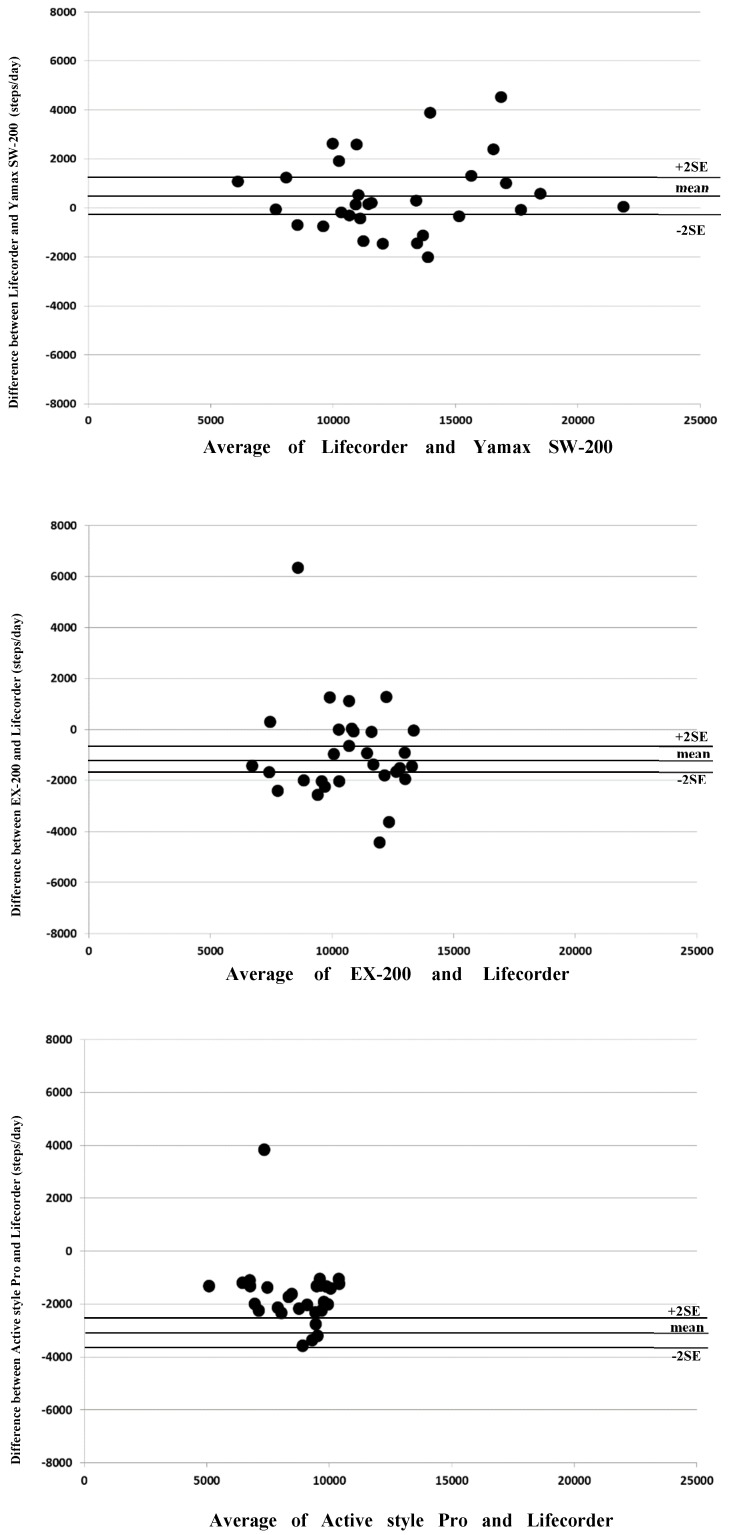
Bland—Altman plot showing the Lifecorder and each pedometer with the mean and the 95% confidence intervals for the bias.

Figure 2 shows the Bland-Altman plots for the various pedometers. The mean difference with Yamax SW-200 was a slightly higher step count compared with Kenz Lifecorder (460 steps·day^−1^). 

Those of Yamasa EX-200 (−1248 steps·day^−1^) and Omron Active style Pro (−2588 steps·day^−1^) were obviously lower compared with Kenz Lifecorder. The limits of agreement (± 2SD) were from +3588 to −2668 steps·day^−1^ for Yamax SW-200, from 2437 to −4933 steps·day^−1^ for Yamasa EX-200 and from −92 to −5084 steps·day^−1^ for Omron Active style Pro.

## 4. Discussion

The purpose of this study was to compare the step counts measured by 4 representative pedometers for primary school children in Japan under free-living conditions. The Kenz Lifecorder provides relatively similar step counts to the Yamax SW-200 during free-living activity (+4.5%), which was compatible with the previous study [12]. The population studied by McClain et al. [12] was 31 fifth-grade boys and girls recruited from a single school located in an urban school district in the southwestern United States. The surrounding environment may be different to some degree, but the mean step counts were not so different, which may have led to similar results.

The accuracy of the Japanese pedometers was determined during the manufacturing process in accordance with the Japanese Industrial Standards (JIS). According to the JIS, the error of the step counts measured by the vibration test is required to be within ±3% (JIS S7200-1993). However, when adult participants simultaneously wore several pedometers under free-living conditions, large differences of around 20–30% or 2000–3000 step counts·day^−1^ were recorded [13]. This is an important issue, because pedometer output is often reported as “steps per day”. The nature of locomotive activities in children is different from that in adults [21]. In addition, the pedometers use different mechanisms. Crouter et al. [22] showed that spring-levered pedometers contain a spring-suspended horizontal lever arm that moves up and down in response to vertical accelerations of the hip. On the other hand, piezo-electric pedometers contain a horizontal cantilevered beam with a weight on one end that compresses a piezo-electric crystal when subjected to accelerations above the sensitivity threshold [21]. 

The present study demonstrated that although the range of differences between Yamax SW-200 and Kenz Lifecorder was not small (from −3588 to +2668 steps·day^−1^), the mean difference was small (−460 steps·day^−1^, ns) and that there is a statistically significant correlation between the two devices (*r* = 0.910, *p* < 0.001), despite the different characteristics of the pedometers to evaluate step counts, as described above. The previous study reported that there was no difference between Yamax SW-200 and Kenz Lifecorder steps (Δ = −200 steps) [12]. Findings of the present study are relatively similar. It is difficult to explain the discrepant results between laboratory settings and free-living conditions. One possible explanation is that some non-ambulatory movements without walking were counted as steps by the spring-levered pedometer (Yamax SW-200). 

The current study showed that compared with Kenz Lifecorder, the Yamasa EX-200 and Omron Active style Pro devices underestimated the step count during free-living conditions by 7.9% and 18.2%, respectively. Step counts were not significantly equivalent between Kenz Lifecorder and each device by the two-one-sided-tests method. Moreover, agreement between Yamasa EX-200 or Omron Active style Pro and Kenz Lifecorder was low (from −5084 to −92 steps·day^−1^ or from −2668 to +3588 steps·day^−1^, respectively). A previous study in adults indicated that accelerometer-based pedometers placed in the pants pocket (in-pocket type of accelerometer) provided valid and reliable step counts measured under prescribed and self-paced walking conditions [23]. On the other hand, Silcott et al. [24] compared the Omron HJ-720ITC piezoelectric pedometer and a spring-levered pedometer in adults during a 24-h period under free-living conditions. The results showed that the Omron HJ-720ITC underestimated the steps per day under free-living conditions more (about 35%) than the Yamax SW-200 (about 20%). Dondzila et al. [25] also compared the New Lifestyles-1000 pedometer with the Omron HJ-720ITC and Kenz Lifecorder EX. The Omron HJ-720ITC recorded a significantly lower number of steps in both young and older adults. The results of the present study are consistent with some of these earlier studies in adults. Silcott et al. [24] considered that this lower count was due, in part, to the presence of a 4-s step filter that contributes to an underestimation of steps accumulated during intermittent activities. In the present study, the error in step counts recorded by Yamasa EX-200 and Omron Active style Pro was larger than that measured in adults. The algorithms to detect steps are not disclosed but differ according to manufacturer and sometimes even in devices made by the same manufacturer. Yamasa EX-200 and Omron Active style Pro may also miss a significant percentage of steps during intermittent lifestyle activities, possibly due to their different acceleration thresholds, filters to avoid counting movements not technically considered walking, and attachment methods. If step counts by very intermittent walking are cancelled more by pedometers, the total step counts decrease. Omron Healthcare has emphasized continuous walking and indicated total minutes of 10 minutes or more continuous walking on the screen of pedometers in addition to total step counts. Actually, Omron Active style Pro provided lower step counts in the present study, which may be caused by the above-mentioned company’s concept. Thus, the influence of different algorithms in devices may be large. A recommendation of pedometer-determined steps per day has been proposed [2]. Evidence on the comparisons between pedometer brands under free-living conditions is therefore necessary to ensure that such a classification scheme for activity status, guidelines or recommendations is meaningful. 

Although the step counts obtained with Kenz Lifecorder and Yamasa EX-200 or Omron Active Style Pro showed large differences, the correlations between these devices were moderate and strong, respectively. This finding indicates that Yamasa EX-200 and Omron Active Style Pro seem to evaluate relative levels of daily step counts in children similar to Kenz Lifecorder. The conversion equations derived in the present study can be used to compare step counts obtained by different devices. For example, conversion from Omron Active style Pro to Kenz Lifecorder assumes an error of ±897 steps·day^−1^ (Table 2).

There were several limitations in the current study, as the populations studied were all generally healthy, and the majority of children were not obese. As a consequence, it may not be appropriate to extend our results to obese populations. For reasons of feasibility, such as built-in memory, Kenz Lifecorder was used to compare the steps measured by the only one pedometer (Yamax SW-200) or the only two uploadable pedometers (Yamasa EX-200 and Omron Active style Pro) during the observation period, because the present study was conducted under free living conditions. Direct observation is a gold standard for pedometer accuracy in free living conditions, so this is not a validation study but a comparison study. However, it is not feasible to observe steps for long time, especially for children at school, home, and community. In future studies, a direct observation needs to be examined in a laboratory setting and in semi-free-living conditions. Previous studies showed that Yamax SW-200 underestimated the actual step counts by almost 10-50% during slow walking speeds under experimental conditions in first-, second-, and third-grade children [8,10]. Moreover, error of the counts recorded at a slow pace by accelerometer-based pedometers, such as Kenz Lifecorder, has been shown to be small, thereby confirming the high accuracy of these devices [10]. Thus, there is limited research on the accuracy of these pedometers in children.

## 5. Conclusions

The daily step counts in free-living conditions of primary school children differed depending on the device used. Step counts measured by Kenz Lifecorder were comparable to those recorded by Yamax SW-200. The correlations between Kenz Lifecorder and Yamasa EX-200 or Omron Active style Pro were moderate and strong, respectively. Our data therefore indicate that the choice of pedometer substantially impacts the measurement of daily step counts. Thus, this study indicates that Kenz Lifecorder is favorably comparable to Yamax SW-200 on a group basis; however, its use as a proxy measure of step counts on an individual basis may be limited. Kenz Lifecorder can be recommended for use in primary school children, because it has a user-friendly software interface and built-in memory. The data from the Kenz Lifecorder are compared with those obtained by the Yamax SW-200, a widely used pedometer in research studies.

## Figures and Tables

**Table 1:** Comparison of daily step counts between pairs of pedometers.

Reference	Pedometer A	n	Reference (steps/day)		Pedometer A (steps/day)		% Difference	
			mean	SD	mean	SD	mean	SD
Yamax SW-200	Lifecorder	30	12425	3562	12885	3751	4.5	13.4
Lifecorder	EX-200	48	13019	3103	11771	2872	−7.9	21.3
Lifecorder	Active style Pro	108	13809	3760	11221	3023	−18.2	10.1

**Table 2:** shows the equations for conversion of step counts between the various devices.

Pedometer A	Pedometer B	Equation for Conversion from Pedometer A to Pedometer B	SEE	Equation for Conversion from Pedometer B to Pedometer A	SEE
Lifecorder	Yamax SW-200	B = 0.8639xB + 1294	1505	A = 0.9581xA + 981	1584
Lifecorder	EX-200	B = 0.7522xA + 1978	1828	A = 0.8776xB + 2689	1693
Lifecorder	Active style Pro	B = 0.7681xA + 615	897	A = 1.1884xB + 474	1116

SEE: standard error of estimate.
